# Control of HIV replication by a novel constitutively active ‘super-PRR’

**DOI:** 10.1186/1742-4690-9-S2-P306

**Published:** 2012-09-13

**Authors:** S Gupta, J Termini, G Stone

**Affiliations:** 1 University of Miami, Miami, FL, USA

## Background

Innate responses are key determinants of the outcome of HIV infection, influencing critical events in the earliest stages of infection. Innate antiviral immune defenses are triggered through the recognition of conserved pathogen associated molecular pattern (PAMP) motifs within viral products by intracellular pattern recognition receptor (PRR) proteins in infected cells. Type I interferons (IFN α and beta) are induced directly in response to viral infection, resulting in an antiviral state for the cell.

## Methods

A ligand independent constitutively active form of PRR adaptor protein (‘super-PRR’) was constructed and tested for antiviral properties. NF-kb and IFN production by construct was tested by Luciferase reporter assays. The construct was transfected into RAW 264.7 cells, analyzed for surface markers and inflammatory cytokines. RT-PCR was performed to analyze the expression of cytokine/chemokine genes. TZM-bl cells were transfected with plasmid expressing ‘super-PRR’, infected with HIV-BaL virus and beta-galactosidase activity was measured.

## Results

The constitutively active ‘super-PRR’ generated 50-fold and 10-fold increase in NF-kb and IFN-beta production respectively as compared to vector alone. There was significant increased expression of CD80, CD86, CD40, CCR7, HLA-DR, secretion of IL-1beta, IL-6, TNF-α and expression of RANTES, MIP-1beta, IP-10 on transfected RAW cells. TZM-bl cells transfected with ‘super-PRR’ after infection with HIV-Bal virus showed significantly reduced replication of virus. In a transwell experiment, 293T cells transfected with the ‘super-PRR’ was sufficient to significantly reduce HIV-1 infection of TZM-bl cells, suggesting soluble factors are involved.

## Conclusion

This constitutively active form of PRR adapter protein induced potent anti-viral innate immune response and prevented infection with HIV. The modulation of innate immunity has potential as a powerful strategy to complement traditional approaches to HIV therapy by protecting cells from viral infection. We are currently constructing lentiviral vector vaccines encoding this ‘super-PRR’ as a method to target this anti-viral response to the site of HIV-1 Infection.

